# Using resilience to predict the effects of disturbance

**DOI:** 10.1038/srep25539

**Published:** 2016-05-05

**Authors:** Stuart Nattrass, David Lusseau

**Affiliations:** 1 University of Aberdeen, Institute of Biological and Environmental Sciences, Aberdeen AB24 2TZ, UK

## Abstract

Animal behaviour emerges from a complex interaction between an individual’s needs, life history strategies and the varying local environment. This environment is increasingly disturbed as human activity encroaches on previously unexposed regions. This disturbance can have different effects on individual animals or populations depending on their behavioural strategies. Here, we examine a means of predicting the resilience of individuals or populations to unanticipated disturbances, and we find that resilience that can be estimated from routinely collected behavioural observations is a good predictor of how rapidly an individual’s expected behaviour is returned to following a perturbation, and correlates strongly with how much population abundance changes following a disturbance.

Life history strategies play a crucial role in shaping the behaviour of animals, setting the priorities of how individuals allocate energy for survival and reproduction. Individual animals can meet these needs by a variety of different behavioral patterns or tactics^1^,^2^,^3^. These behavioural patterns are also shaped by environmental constraints and opportunities, such as food availability or predation risk. We can therefore envisage that different behavioural patterns will evolve under different environmental pressures^3^. For example, if individuals rely on pulsed food resources, those who are able to have prolonged foraging bouts and greater abilities to store energy during periods when food is scarce would be advantaged^4^.

The rapidly expanding anthropogenic landscape presents novel selection pressures to which animals have to adapt^5^. Any individuals unable to adapt to these novel pressures may suffer reductions in survival or reproductive success. Therefore, these indirect effects can create a conservation challenge, particularly for those populations and species commonly exposed to human interference. Here, we suggest a simple method to predict which behavioural strategies may be most affected by anthropogenic behavioural perturbations.

Animal behavior can be formalized as emerging from the interaction between the ecological landscape, the individual’s experiences, their needs and fitness and their behavioural strategies. This is not novel and was first formalised by McNamara and Houston when they described state-dependent life histories^3^. The ecological landscape that an individual experiences is not constant, but varies in time. While many fluctuations in the environment have been anticipated by the behaviour that has evolved in the animal, human presence may provide several novel selection pressures on an individual’s behavioural strategy. For example, many studies show that animals try to avoid human disturbances such as vessel traffic, forgoing their current activities to move away from the disturbed area^6^,^7^,^8^. Repeated elicitation of this response can affect the individual’s condition as time spent avoiding human activity is taken away from activities such as foraging, which can then impact on their survival or reproductive success if an individual has difficulty compensating for these lost opportunities^9^,^10^. Therefore, the ability of an individual to adapt their behaviour, or recover quickly from a disturbance, within an evolved strategy may be crucial in determining its demographic contributions. One way we can measure this ability to adapt to disturbances is the concept of resilience.

Resilience was first introduced to the ecological literature by Holling, as a measure of the persistence of an ecosystem or series of populations^11^, and has since been defined variably as the ability of a system to withstand perturbation^12^, or the speed at which a system will recover^13^. Here, we consider resilience as capturing the time to recover from a perturbation, with one key adjustment. While resilience has been used widely to examine how diverse communities respond following a disturbance, to our knowledge, the concept has never been applied to assessing the response of a single individual to a behavioural disturbance by anthropogenic forces. Thus, the measure of resilience presented here describes how long the effects of disturbance events last before an individual (or the average animal in a population) returns to its expected behaviour.

We examine the resilience of behavioural strategies determined by physiological effects such as the efficiency with which an individual converts food to improved condition such as added body weight, and depending on fitness constraints and environmental variability using a simple model where an animal can vary its level of feeding. This model can be represented as dynamic functions as in

equation (1) used to determine an individual’s response to disturbance, where disturbance is considered to be any anthropomorphic pressure that affects behaviour or environmental quality. Increased feeding levels will improve the animal’s condition, while depleting the quality of the environment on which it feeds. As the condition of the animal improves, its desire to feed will decline; hence it will feed less. Finally, the quality of the environment is such that when it is poor, it is likely to decline further, while a good environment will continue to be good quality; varying the parameter that determines the strength of this interaction allows us to vary how the environment itself responds to changes in environment. This set of biologically grounded functions gives the ordinary differential equation model presented in

equation (1) (see Methods for further details). Here, we consider condition as a proxy for individual fitness, as condition has been linked to improved survival^14^,^15^,^16^ and reproduction^17^,^18^,^19^,^20^,^21^ in a number of species.

We consider two different ways in which feeding can increase condition; first, a simple linear relationship where doubling the amount of food eaten will increase condition twice as much (Model 1), and second, the scenario where higher food intakes result in decreasing fitness returns, such that eating double the food will not increase condition by twice as much (Model 2). We randomly generated 10^6^ sets of parameters (Supplementary material) that are used to calculate and examine the resilience of different types or equilibria, both attracting and otherwise, and show how the concept of resilience can predict the response of the animal to a disturbance event. Finally, we expand this individual model to a population model. We consider a model that explicitly takes into account the population size (Methods), and see how a resilience measure that includes effects of the population size affects both the recovery time of the average condition in the population and the population size itself. Further, we demonstrate that even with incomplete knowledge of a system, it may be possible to accurately assess how resilient to disturbances a population is.

## Results

**Figure 1:**
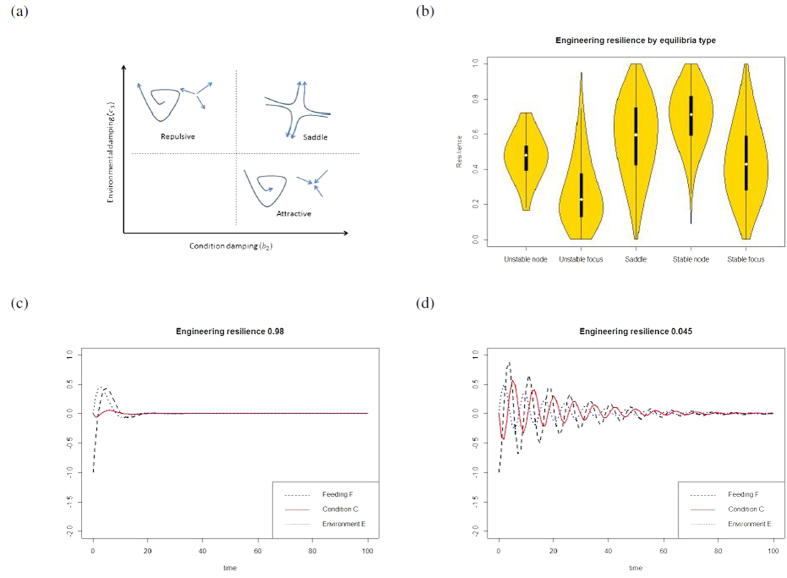
How resilience can predict effects of disturbance. (**a**) Stable behavioral systems are more likely to occur when the environment returns to the unpredated stable point more slowly (lower values for *c*_3_). However, if damping on the individual’s condition is reduced, repulsive equilibria are more common. (**b**) The engineering resilience, or recovery time, for Model 1, where condition increases linearly with feeding, grouped by equilibrium type. The shading gives the density function for the resilience, while the white dot shows the mean and the thick, black lines show the 50% confidence intervals, while the narrower lines are the 95% confidence intervals. (**c**,**d**) Model time series for systems with similar stability (stable foci) but different resilience values when the system is perturbed by decreasing the feeding rate of the individual from the equilibrium value of 0 to −1. The system with the higher engineering resilience showed a faster return to the equilibrium.

There is a single equilibrium at the origin when condition gain is linear (Model 1, equation (2)). However, the type of equilibrium changes depending on the rate of change of the variables. The equilibrium at the origin can be either attracting, repulsive or a saddle point (Fig. 1a). Those equilibria that are attracting are more present in environments with longer time to prey equilibrium (lower value for the parameter *c*_3_), than saddles or repulsive equilibria. In contrast, repulsive equilibria are present when the condition of an individual can change quickly (lower value of *b*_2_, the parameter determining how much damping there is on the condition of the individual), that is; models with unstable fixed points show less damping on condition, representing situations when condition changes rapidly following feeding success.

Stable fixed points have higher resilience than the equivalent repulsive fixed points, but this measure is not significantly higher for stable equilibria than for saddles (Fig. 1b). Further, even among stable equilibria, variations in resilience can affect response to a disturbance (Fig. 1c,d). When the disturbance event causes the environment to decline from the equilibrium at the origin, models with high resilience return to the fixed point faster than models with lower return rates, and condition is less affected, demonstrating that resilience can be an effective measure for considering the consequences of disturbance.

When higher feeding rates show diminishing returns in their effect on condition (Model 2, equation (3)) then the system has two possible equilibria. As in the linear model, there is a fixed point at the origin, where feeding rates, condition and environmental quality are all low. However, there is also a second fixed point, in 71% of simulations, which has positive feeding rates, condition and environment. This fixed point therefore shows a behavioural strategy that increases the condition of the individual, which may have significant effects on survival or reproductive output.

**Figure 2:**
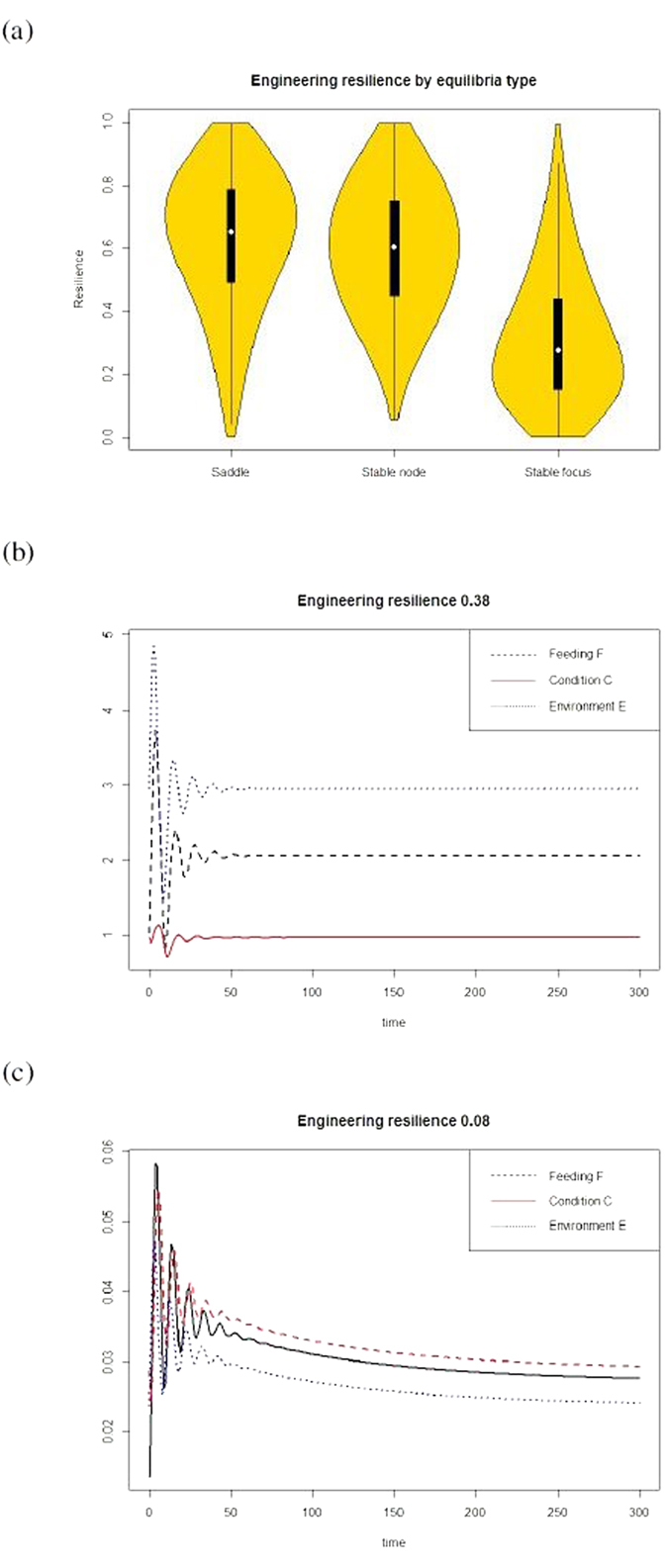
Resilience in a non-linear model. (**a**) The engineering resilience, or recovery time, for Model 2, where condition increase tapers off at high feeding levels, grouped by equilibrium type. The shading gives the density function for the resilience, while the white dot shows the mean and the thick, black lines show the 50% confidence intervals, while the narrower lines are the 95% confidence intervals. (**b,c**) Model time series for systems with similar stability (stable foci) but different resilience values when the system is perturbed by decreasing the rate of feeding from the equilibrium value to half the equilibrium value. The system with the higher engineering resilience showed a faster return to the equilibrium.

When this second fixed point gives an increased condition for the individual, it can represent either a saddle or an attracting equilibrium. As with the linear model we can compare the parameter combinations that give these different equilibria type, and we find that this analysis returns the same result as for Model 1, where attracting equilibria are more prevalent in less volatile environments, where return to the non-predated fixed point is slower (lower values of *c*_3_). Further, we also replicate the findings from the linear model when we consider the resilience of attracting foci versus attracting nodes, where we find that foci have lower resilience than stable nodes (Fig. 2a).

**Figure 3:**
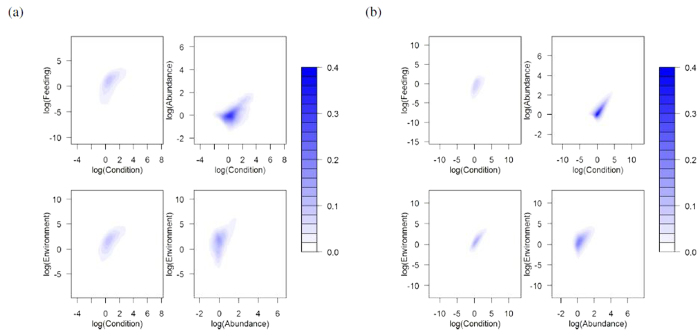
Density distribution of stable states. Conditional density distribution of equlibria for stable states (**a**) attractive nodes, n = 3516, and (**b**) attractive foci, n = 307281); color intensity corresponds to the density of those stable states.

**Figure 4:**
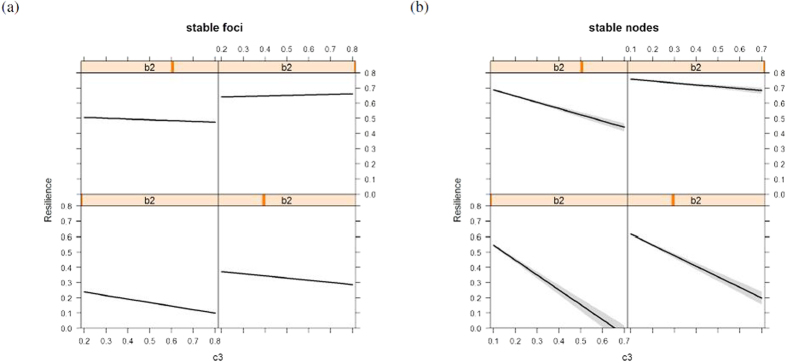
Estimating resilience in data poor conditions. In systems where data is poor, resilience, and thus time to return after a perturbation, can be estimated from first principle by understanding the physiology of the species (the rate at which individuals are able to change condition *b*_2_) and environmental productivity dynamics (*c*_3_). Predicted resilience of population model (equation 4) for stable foci (**a**) and stable nodes (**b**) depending on condition loss rate (*b*_2_) and environment replenishment rate (*c*_3_). Lines are predictions based on generalised linear models with 95% confidence intervals (gray bands); panels represent increasing values of *b*_2_ (from bottom left to top right. Bottom left = 0.2, bottom right = 0.4, top left = 0.6 and top right = 0.8).

When population size is explicitly accounted for in the model (equation 4), results are similar to the models that focus on a single individual. Stable, attracting equilibria occur in environments where the recovery rate of the environment, for example prey population, is much lower (lower values of *c*_3_), while repulsive equilibrium points are found in systems where the damping on average condition is low (low *b*_2_) (Fig. 1(a)). Resilience values are also lower in unstable systems, while among stable equilibria, foci have lower resilience than nodes (*F*_1,310795_ = 5023, *p* < 0.00001, Fig. 3 presents the coordinates of those equilibria along the 4 dimensions of the system). Further, we find that even within the stable foci (31% of all simulations), environmental recovery rate and condition damping are crucial in determining the resilience of a population. Environmental recovery *c*_3_ and condition damping *b*_2_ can explain 68% of the variation in resilience values (Fig. 4a, *F*_3,307277_ > 2000, *p* < 0.00001). They can also explain 36% of resilience in stable nodes (Fig. 4b, *F*_3,3512_ = 664, *p* < 0.00001). In both types, resilience decreases as the environmental recovery rate increases, however this environmental effect is dampened by the rate at which individuals lose condition (Fig. 4). Hence, resilience can be estimated from first principle, in data scarce conditions, with a basic understanding of the physiology of the species (the rate at which it is able to change condition) and the productivity dynamics of its environment. Species that are able to change condition faster will be more resilient.

**Figure 5:**
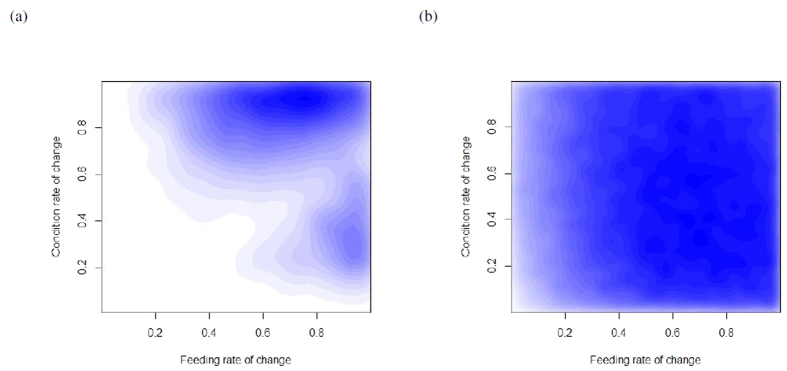
Estimated condition (*b*_2_) and feeding (*a*_1_) rate of change for attractive nodes (**a**) and foci (**b**). Nodes have a bimodal distribution along feeding and condition change, while foci do not.

These simulations showed that key system parameters differed between stable equilibrium types. Of particular interest is the relationship between feeding rate of change (*a*_1_) and condition rate of change (*b*_2_). These change along the axis of capital-income breeding strategies, with capital breeders having a slow condition rate of change and a higher feeding rate of change; while income breeders tend to have a faster condition rate of change. Attractive nodes seem to assort themselves into two groups along this axis (Fig. 5a), while attractive foci did not (Fig. 5b).

**Figure 6:**
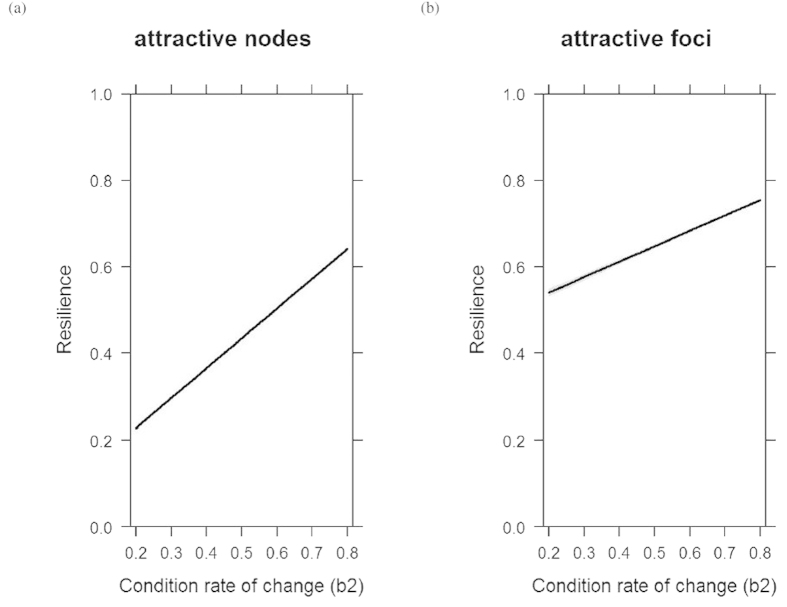
Effect of condition rate of change on resilience. The influence of condition rate of change (*b*_2_) on system’s engineering resilience. Predicted value from a fitted general linear model, including 95% confidence interval (grey band).

For all stable systems, the condition rate of change (*b*_2_) influences the resilience of the systems (Fig. 6). In other words, the higher the damping on condition, the more resilient it is. So we expect life history strategies selecting for slow change in condition to be more resilient to perturbations.

### Simulated perturbations

**Figure 7:**
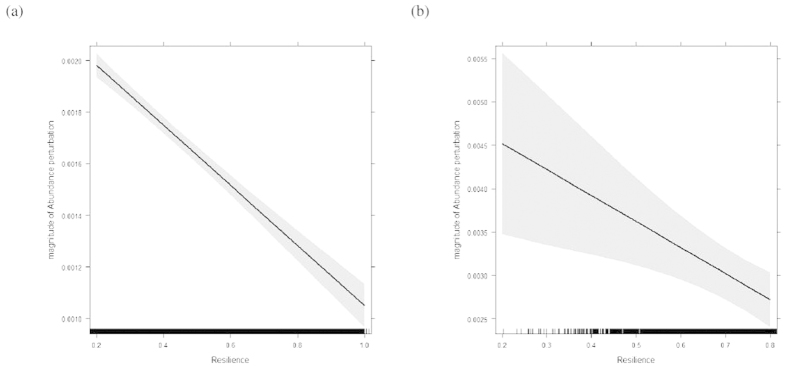
Predicted effect of resilience on population abundance change. Predicted effect of resilience on the magnitude of abundance change in response to an environmental perturbation, grey band is 95% confidence interval, for attractive foci (**a**) and nodes (**b**). Note, the difference in the y-axes of the plots, on average nodes were more perturbed than foci.

This work provides predictions for the way each of those 1,000,000 systems should respond to perturbations. We simulated an environmental perturbation by decreasing the value of E for each stable systems by 10%, hence decreasing food availability. Results were qualitatively similar when feeding rates were decreased instead, simulating foraging disruption instead of decreased food availability. We then tracked the magnitude of the effect this environmental perturbation had on the population abundance as well as the time it took the population abundance to recover to the stable N. The environmental perturbation had less of an effect on more resilient attractive foci systems (*F*_1,306292_ = 267.88, *p* < 0.0001, Fig. 7a) and more resilient attractive node systems (*F*_1,3512_ = 9.45, *p* = 0.002, Fig. 7b). In both instances, the same perturbation had less population-level impact when the system was estimated to be more resilient. However, the system resilience did not predict the time it took abundance to recover from the perturbation. This system-level resilience measure was not a good proxy for variable-level predictions on recovery time.

## Discussion

Two main conclusions emerge from this simulation work. Firstly, the dynamics of the environments in which populations live is a key factor in stability; environment that change slowly are more likely to confer the foundations for stable systems. Those environments are more likely to provide the means for populations to recover from perturbations. Secondly, The resilience of those populations to perturbations, which encompasses both the magnitude of the perturbation’s effect as well as the time it will take an individual within the population to recover, will depend on the rate at which the condition of individuals will change. Slow condition change will increase the resilience of the populations. The transient dynamics around the equilibria will affect resilience as well, attractive node are less resilient than attractive foci.

The concept of resilience can be used to predict the ability of individuals with different behavioural strategies to compensate for disturbances. This represents their ability to adapt their behaviour quickly in order to minimize the potential that these disturbances will have consequences for their demographic contributions. We have shown that we can predict the individual behavioral strategies that will reduce the effects of a disturbance. To expand this out to a population level effect, we can consider two possible routes. Firstly, if each run of the individual models presented here represents an individual animal with the behavioral responses to the environment, then a collection of individuals may be represented by multiple sets of parameters used in the same model framework. From this, the distribution of resilience in the population can be easily calculated. A population wide measure can then easily be determined, considering the average resilience of the individuals or some threshold value for which a given percentage of the population show resiliences larger than (for example, 50% of the population would return higher values than the median resilience). This approach to population level measurement of resilience may miss compounding effects in social species, where group membership trade-offs are considered by individuals for their actions. We anticipate that this simpler approach will prove appropriate to species where individuals spend a significant proportion of time as independent individual.

Second, we consider a measure of resilience that incorporates the effects of population size and it’s interactions with the average condition and environment explicitly, rather than post-hoc. Within this model, we demonstrate that resilience can be estimated using only information on the environment and the condition rate of change of the studied species. The type of data to develop behavioural models to estimate resilience is now readily available for some populations^22^, although proxies for feeding rates are often necessary. Even when full parameterisation of the system is not possible, we demonstrate that resilience of a population to a disturbance is tied closely to the rate at which condition and environment respond to themselves - in systems where environmental recovery (such as the intrinsic growth of a prey population) and condition dynamics can be estimated, from data or from first principles, resilience can also be estimated with a degree of confidence. We can therefore use this approach to prioritize conservation efforts on areas or populations that contain more vulnerable individuals. Further, we believe this measure can be used to inform risk assessments of populations where disturbances events may be small but frequent^5^,^9^.

The models considered here assume a stable, fixed environment in space and time, given the absence of the studied individuals or species. Clearly, this assumption is likely to be invalid, especially in the presence of climate change effects. However, for short-term disturbances the temporal aspect of resource availability is unlikely to alter dramatically, unless the prey items also exhibit avoidance activity following human disturbances. Spatial variation may persist over the course of a short disturbance, and future expansions to the current framework should include spatial variation in resource availability. However, spatial variation in prey availability for many species may be over small scales compared to the home range or movement abilities of the focal species, and in those cases, average resource availability should provide a fair indication of resources throughout the range.

While this resilience measure can be effective at discerning how population abundance will change after a disturbance, and the explicit population model we examine here shows a strong correlation between resilience and how much the population size will decrease following a disturbance, it may not be able to predict whether an individual animal has been disturbed to such an extent that it may never recover. We still lack a strong predictor of how far an individual or population can be pushed before their behaviour is permanently altered, which may affect their demographic contributions (state shift in life histories)^3^. Despite this, resilience can provide a simple framework to inform the propensity for population consequences to emerge from behavioral disturbances, thus allowing to prioritize conservation efforts to be focused on the populations most vulnerable to sub-lethal impacts.

## Methods

The two models examined here are given by the set of ordinary differential equations


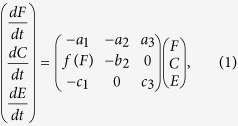


where *F* is the feeding rate of a individual, *C* is the condition of that individual (here used as a proxy for individual fitness), and *E* is the quality of the environment in which the individual finds itself. Environment quality can be interpreted as resource richness, ensuring that low quality environments reduce the feeding rate of an individual when compared to higher quality environments. The parameter *a*_3_ indicates how much feeding increases with an improvement in resource availability. Parameter *a*_2_ is the scale at which a well-conditioned animal will scale back it’s feeding, whether for physiological reasons (for example, marine mammals with high fat stores become more buoyant, ensuring prey in deep waters become relatively harder to acquire) or via satiation, such as in reptiles who eat very seldomly. *a*_1_ indicates damping on the feeding rate itself - as an individual must also perform other activities, a high feeding rate is unsustainable, so when the current feeding rate is high the individual will slow down, while low current rates will drive the individual to increase the time spend foraging. Larger *a*_1_ will increase the non-feeding demands of an individual, resulting in a larger decrease in feeding rate when it has previously been high.

The first model, where condition increases linearly with feeding, is defined by


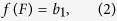


while the non linear model, where condition shows a lower response to feeding rate increases when feeding is higher initially, is given by


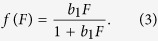


Parameter *b*_1_ can be considered as the feeding efficiency of an individual, determining how well food intake is translated into improved condition, while parameter *b*_2_ indicates that positive condition will decline with time when no food is added, and can thus be interpreted as a measure of metabolism speed.

Feeding rates will reduce the abundance of resources in the environment, and *c*_1_ indicates how quickly this will occur. The higher *c*_1_ is, the more efficient a forager the individual is, and therefore the larger the decrease in environmental quality for a fixed feeding rate. Finally, *c*_3_ indicates that the environment, in the absence of the considered individuals, will find a fixed value (such as the carrying capacity of a prey item). Therefore, in the absence of feeding pressure environments with higher resources or quality will decline, and low quality environments will increase. Higher values of *c*_3_ indicate faster reversion to this fixed value.

For the final model presented here, we include a population size variable *N* in the system of differential equations, to allow us to consider how density dependent interactions will affect the calculation of resilience. Here, the structure of the non linear model 2 above is used, with some alterations. Note that the condition and feeding rate parameters are considered as population averages, rather than measures of a specific individual. The model is then given by


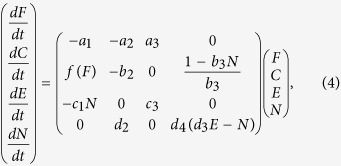


where the values of the parameters *d*_*i*_ are drawn from a uniform distribution over the interval [0, 0.01], two orders of magnitude smaller than the other parameters, to indicate the slower time scale changes in population occur over. The parameter *d*_2_ indicates that a group with high average condition will be expected to have greater survivorship and reproductive output, and therefore the population *N* will grow faster. The population size at which density dependence becomes detrimental is determined by *d*_3_, with the carrying capacity of an environment with quality *E* given by *d*_3_*E*, while *d*_4_ determines the strength of the density dependent effects, with a higher *d*_4_ ensuring density dependent effects are stronger. Note that high population will lead to reductions in condition as competition increases (*b*_3_).

Mathematically, resilience can be calculated from the Jacobian of a behavioral model, the matrix of partial derivatives of the model, which is determined by the model parameters. Resilience, formally ‘engineering resilience’, is given by the maximum absolute value of the real parts of the Jacobian’s eigenvalues, *max_*i*_*|*Re*(*λ*_*i*_)| where *λ*_*i*_ is the *i*-th eigenvalue of the Jacobian **J**.

## Additional Information

**How to cite this article**: Nattrass, S. and Lusseau, D. Using resilience to predict the effects of disturbance. *Sci. Rep*. **6**, 25539; doi: 10.1038/srep25539 (2016).

## Supplementary Material

Supplementary Information
